# Effects of Triiodothyronine on Human Osteoblast-Like Cells: Novel Insights From a Global Transcriptome Analysis

**DOI:** 10.3389/fcell.2022.886136

**Published:** 2022-06-17

**Authors:** Bruna Moretto Rodrigues, Lucas Solla Mathias, Igor de Carvalho Deprá, Sarah Santiloni Cury, Miriane de Oliveira, Regiane Marques Castro Olimpio, Maria Teresa De Sibio, Bianca Mariani Gonçalves, Célia Regina Nogueira

**Affiliations:** ^1^ Department of Internal Medicine, Medical School Botucatu, São Paulo State University (UNESP), Botucatu, Brazil; ^2^ Department of Structural and Functional Biology, Institute of Biosciences, São Paulo State University (UNESP), Botucatu, Brazil

**Keywords:** osteobalst, triiodothyronine, BMP—smad signaling pathway, RNA-seq, TGF-beta signaling pathway

## Abstract

**Background:** Thyroid hormones play a significant role in bone development and maintenance, with triiodothyronine (T3) particularly being an important modulator of osteoblast differentiation, proliferation, and maintenance. However, details of the biological processes (BPs) and molecular pathways affected by T3 in osteoblasts remain unclear.

**Methods:** To address this issue, primary cultures of human adipose-derived mesenchymal stem cells were subjected to our previously established osteoinduction protocol, and the resultant osteoblast-like cells were treated with 1 nm or 10 nm T3 for 72 h. RNA sequencing (RNA-Seq) was performed using the Illumina platform, and differentially expressed genes (DEGs) were identified from the raw data using Kallisto and DESeq2. Enrichment analysis of DEGs was performed against the Gene Ontology Consortium database for BP terms using the R package clusterProfiler and protein network analysis by STRING.

**Results:** Approximately 16,300 genes were analyzed by RNA-Seq, with 343 DEGs regulated in the 1 nm T3 group and 467 upregulated in the 10 nm T3 group. Several independent BP terms related to bone metabolism were significantly enriched, with a number of genes shared among them (FGFR2, WNT5A, WNT3, ROR2, VEGFA, FBLN1, S1PR1, PRKCZ, TGFB3, and OSR1 for 1nM T3; and FZD1, SMAD6, NOG, NEO1, and ENG for 10 nm T3). An osteoblast-related search in the literature regarding this set of genes suggests that both T3 doses are unfavorable for osteoblast development, mainly hindering BMP and canonical and non-canonical WNT signaling.

**Conclusions:** Therefore, this study provides new directions toward the elucidation of the mechanisms of T3 action on osteoblast metabolism, with potential future implications for the treatment of endocrine-related bone pathologies.

## Introduction

The skeletal system undergoes intense metabolic activity, maintaining a continuous process of bone remodeling through the action of bone cells. Osteoblasts originate from mesenchymal stem cells and are responsible for the synthesis of the bone extracellular matrix, deposition and mineralization of new bone, thereby promoting bone formation (Hadjidakis and Androulakis, 2006; Cawthray et al., 2017). Additionally, osteoblasts control bone remodeling by modulating osteoclastogenesis and bone resorption by the osteoclasts (Hadjidakis and Androulakis, 2006; Feng and McDonald, 2011; Boyce et al., 2012; Hayden et al., 2014).

Thyroid hormones (THs) act as regulators of the bone remodeling process and influence formation of the skeletal system (Hadjidakis and Androulakis, 2006; Boyce et al., 2012; Cawthray et al., 2017). Osteoblasts are known to express nuclear receptors for THs, namely, thyroid hormone receptor beta (*THRB*) and alpha (*THRA*) (Abu et al., 1997; Kim and Mohan, 2013). The THs triiodothyronine (T3) and thyroxine (T4) are especially essential for bone development and maintenance (Bassett et al., 2003; Straub, 2014), as changes in their levels may affect bone metabolism and cause abnormalities, such as changes in the bone mineral density (Waung et al., 2012; Kim and Mohan, 2013; Wojcicka et al., 2013).

Although T3 in particular is known to play an important role in osteoblastogenesis (Klaushofer et al., 1995; Waung et al., 2012; Kim and Mohan, 2013; Wojcicka et al., 2013; Olímpio et al., 2019), its exact biological and molecular mechanisms of action have not been fully elucidated (Harvey et al., 2002; Waung et al., 2012; Kim and Mohan, 2013; Pascual and Aranda, 2013). Therefore, in this study, we aimed to evaluate the effects of different T3 doses on gene expression in osteoblast-like cells, differentiated from human adipose-derived mesenchymal stem cells (hASCs), through a global transcriptome analysis, using RNA sequencing (RNA-Seq) techniques. Overall, our data provide innovative information that adds to existing knowledge about bone development and will help toward clarifying the role that T3 plays in the pathophysiological mechanisms of bone diseases.

## Materials and Methods

### Cell Culture

This study was approved by the Ethics Committee of the Botucatu Medical School, São Paulo State University (UNESP; Approval No.3216-2009). Primary cultures of the previously characterized model of hASCs (Olimpio et al., 2018) from three donors were provided by the Experimental Research Unit (Unipex) cell bank of UNESP. The methods used to culture the hASCs and to induce their differentiation into osteoblast-like cells were carried out as previously described (for details, see Olimpio et al., 2018). In brief, hASCs were isolated from subcutaneous adipose tissue obtained from three patients undergoing abdominoplasty, up to 50 years of age with normal erythrocyte sedimentation rate (ESR). Subcutaneous adipose tissue samples were then submitted to enzymatic digestion. The isolated hASCs were plated at a density of 2 × 10^5^ in a T25 flask, and grown in a complete medium, defined as Dulbecco’s modified Eagle medium (DMEM), containing 10% fetal bovine serum (FBS) with 1% penicillin-streptomycin and 0.1% gentamicin (10 mg/ml; Invitrogen). Upon reaching 70% confluency, cells were trypsinized and transferred to a T75 flask for cell expansion. All cell cultures were maintained at 37°C in a humidified atmosphere with 5% CO_2_. For hASC differentiation into osteoblasts, cells were kept in complete DMEM supplemented with 0.1 μM dexamethasone (Sigma-Aldrich), 50 μM ascorbic acid (Sigma-Aldrich), and 10 mm β-glycerophosphate (Sigma-Aldrich) for 16 days. The resulting osteoblast-like cells were then treated with either 1 nm or 10 nm T3 for 72 h. Osteoblast-like cells grown in the absence of T3 were used as controls.

### RNA Sequencing and Bioinformatics

Total RNA was extracted from the osteoblast-like cells using the TRIzol reagent method (Invitrogen, Carlsbad, CA, United States). The cDNA library preparation, RNA sequencing, and bioinformatics analysis were carried out using previously described methods (de Oliveira et al., 2020). DEGs were classified as being upregulated or downregulated on the basis of fold-change (FC) values > 1.5, with *p* < 0.05. The Gene Ontology (GO) enrichment analysis for biological process (BP) terms was performed with the clusterProfiler R package, using a p-value-adjusted false discovery rate and a *p*-value of < 0.05. Pre-analysis of the GO data was performed, and terms distant from the area of interest were excluded. For the enriched GO terms grouped by similarity (0.7) representation, interactive graphs and TreeMaps were created using REVIGO (http://revigo.irb.hr/) (Supek et al., 2011). DEGs were also analyzed with respect to their protein-protein interactions (PPI) using STRING. Interaction maps were generated considering the following levels of evidence: homology, coexpression, experimentally determined interactions, database-annotated interactions, and text mining. Enriched GO terms were assessed by having an FDR < 0.05.

## Results

### Characterization of Osteoblast-Like Cells

The RNA-Seq analysis revealed the expression patterns of 16,296 genes in the two groups of T3-treated osteoblast-like cells. Of the 10 most expressed genes from this data set, four encoded bone markers: fibronectin 1 (*FN1*), osteonectin (*SPARC*), and collagen type I alpha 1 and 2 chains (*COL1A1* and *COL1A2*). In agreement with other published results, the presence of the nuclear receptors *THRA* and *THRB* was also noted, with the former being more abundant in these cells. Additionally, in a previous study conducted by our research group (Olímpio et al., 2019), the presence of genes encoding other bone markers was observed: osteocalcin and alkaline phosphatase proteins, matrix proteins for bone mineralization, and receptor activator of nuclear factor kappa-Β ligand (*RANKL*).

### Transcriptional Regulation by the T3 Treatments

**FIGURE 1:**
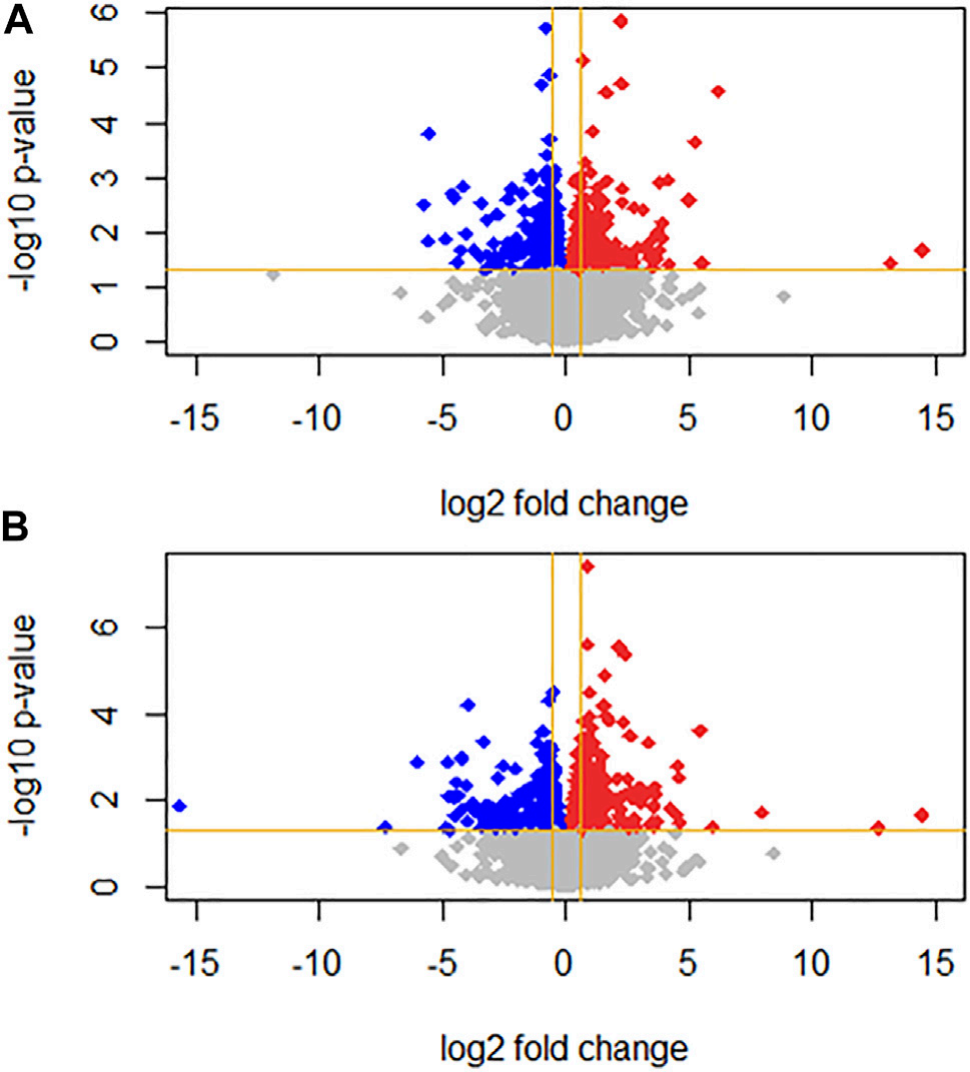
Volcano plots representing gene expression log2 fold change (FC; x axis) and *p*-value (y axis) for **(A)** 1 nm T3 and **(B)** 10 nm T3. Grey dots represent genes with non-significant FC; up- and downregulated genes are represented as red and blue dots, respectively.

**FIGURE 2:**
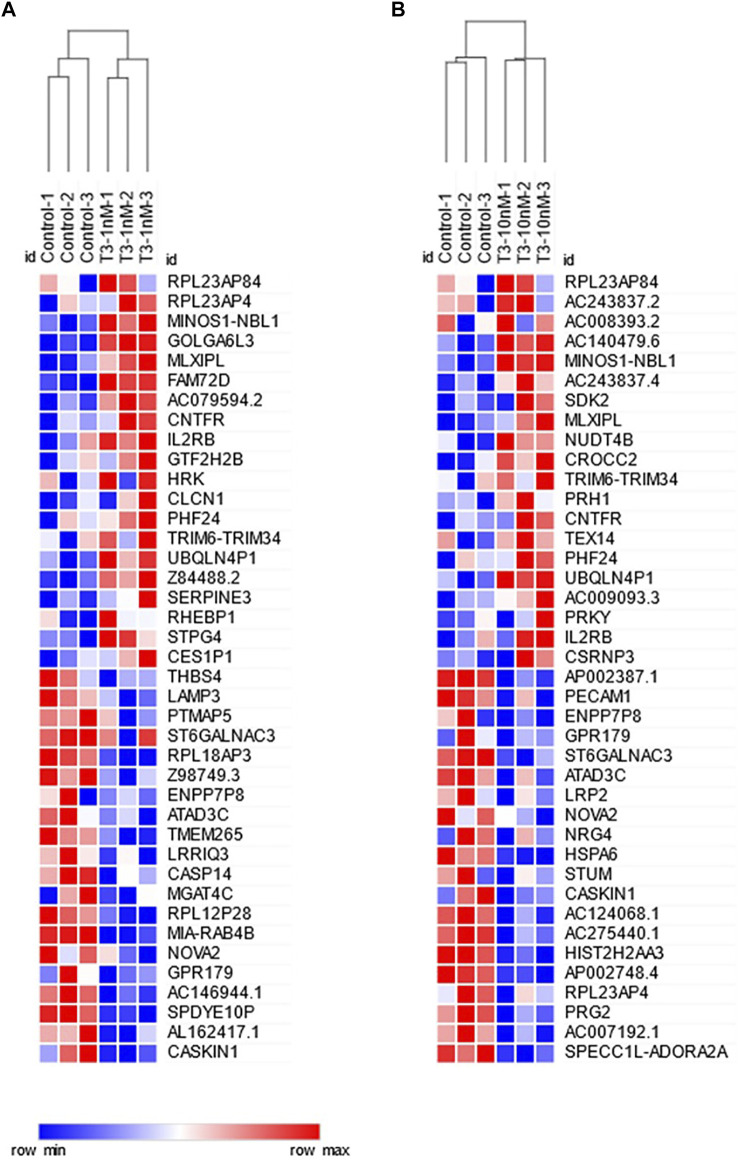
Heatmaps showing the 20 most upregulated (upper half) and 20 most downregulated (lower half) genes for **(A)** 1 nm T3 and **(B)** 10 nm T3. Samples (columns) and genes (rows) are hierarchically clustered by mean Euclidean distance.

**FIGURE 3:**
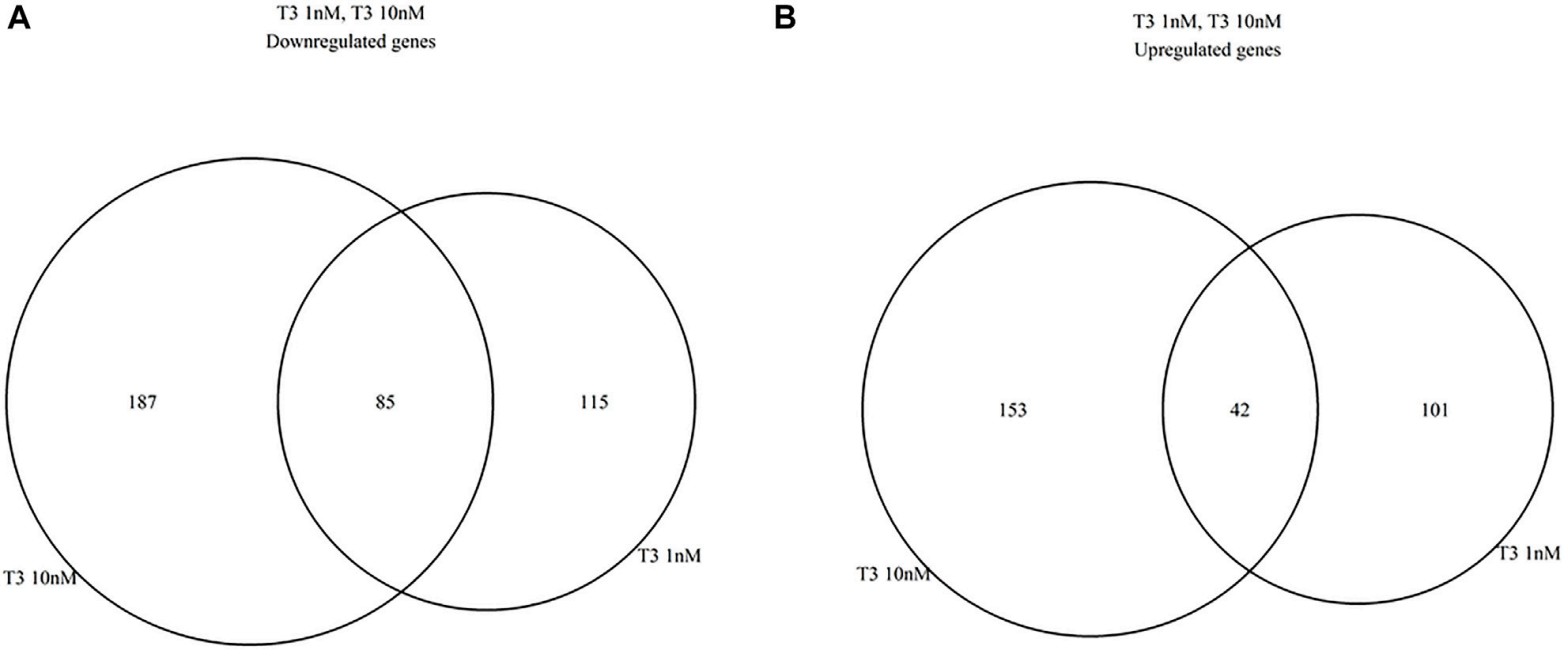
Venn diagrams summarizing similarities between 1 and 10 nm T3 expression profiles, relative to the Control group; **(A)**, dowregulated genes, **(B)** upregulated genes.

Differential gene expression was analyzed between the T3-treated and control (non-treated) groups (Figures 1, 2, and Supplementary Material). For the 1 nM T3 group, 343 differentially expressed genes (DEGs) were identified, of which 200 were upregulated (58%) and 143 were downregulated (42%). For the 10 nm T3 group, 467 DEGs were identified, of which 272 genes were upregulated and 195 were downregulated (also 58 and 42%, respectively). There was an overlap of roughly 20% among genes regulated by both doses (Figure 3) and, importantly, no gene was altered in opposite directions by one T3 dose compared to the other (not shown).

### Gene Ontology Analysis—ClusterProfiler

For 1nM T3, eight and 56 GO biological process (BP) terms were significantly enriched in the up- and down-regulated gene sets, respectively; after manually filtering the terms, we found 11 terms were relevant to the study, all enriched in the down-regulated gene set (Table 1). For the 10 nm T3 group, 49 BP terms were enriched for up-regulated genes, but none reached significance for down-regulated genes. Among the significant terms, the majority was related to embryonic development and none has apparent relation to osteoblast biology; the complete overrepresentation analysis results are presented in the Supplementary Material. The REVIGO tool, which summarizes GO terms on the basis of semantic similarity to reduce redundancy, was used to simplify the results from 1 nm T3 treatment and to clarify the BPs affected (Supek et al., 2011); after analyzing the BP terms with REVIGO, TreeMaps was used to group 11 BP terms into six main terms for the downregulated genes of the 1 nm T3 (Table 1). The main genes involved in the enriched BP terms (Table 2) are examined in the Discussion.

**TABLE 1:** GO terms significantly enriched for genes downregulated after 1 nm T3 treatment, manually filtered for relevance to osteoblast biology. The 11 terms were hierarchically grouped under six main terms (bold) using TreeMaps.

Description	GO ID	Gene count	Gene ratio	Bg ratio	Adj. *p*-value
**mesenchymal cell proliferation**	GO:0010463	4	4/105	44/18670	0,027
stem cell proliferation	GO:0072089	5	5/105	120/18670	0,036
positive regulation of mesenchymal cell proliferation	GO:0002053	3	3/105	25/18670	0,031
**regulation of cell morphogenesis involved in differentiation**	GO:0010769	8	8/105	301/18670	0,031
cell fate commitment	GO:0045165	7	7/105	271/18670	0,039
**ossification**	GO:0001503	9	9/105	398/18670	0,031
**cellular response to retinoic acid**	GO:0071300	4	4/105	69/18670	0,036
positive regulation of Wnt signaling pathway	GO:0030177	6	6/105	179/18670	0,036
positive regulation of chemotaxis	GO:0050921	5	5/105	135/18670	0,044
**protein kinase C signaling**	GO:0070528	4	4/105	29/18670	0,022
**peptidyl-tyrosine phosphorylation**	GO:0018108	9	9/105	363/18670	0,029

Gene count, number of differentially expressed (DE) genes associated with the GO term; gene ratio, associated genes/total DE genes; Bg ratio, number of associated genes/total background genes (all genes annotated in the Gene Ontology Consortium database); Adj *p*-value, false discovery rate-adjusted *p*-value.

**TABLE 2:** Main downregulated genes contributing to enriched GO terms, in the 1 nm T3 group.

FGFR2 fibroblast growth factor receptor 2
WNT5A Wnt family member 5A
WNT3 Wnt family member 3
ROR2 receptor tyrosine kinase like orphan receptor 2
VEGFA vascular endothelial growth factor A
FBLN1 fibulin 1
S1PR1 sphingosine-1-phosphate receptor 1
PRKCZ protein kinase C zeta
TGFB3 transforming growth factor beta 3
OSR1 oxidative stress responsive kinase 1
AREG amphiregulin

### Gene Ontology Analysis—STRING

**FIGURE 4:**
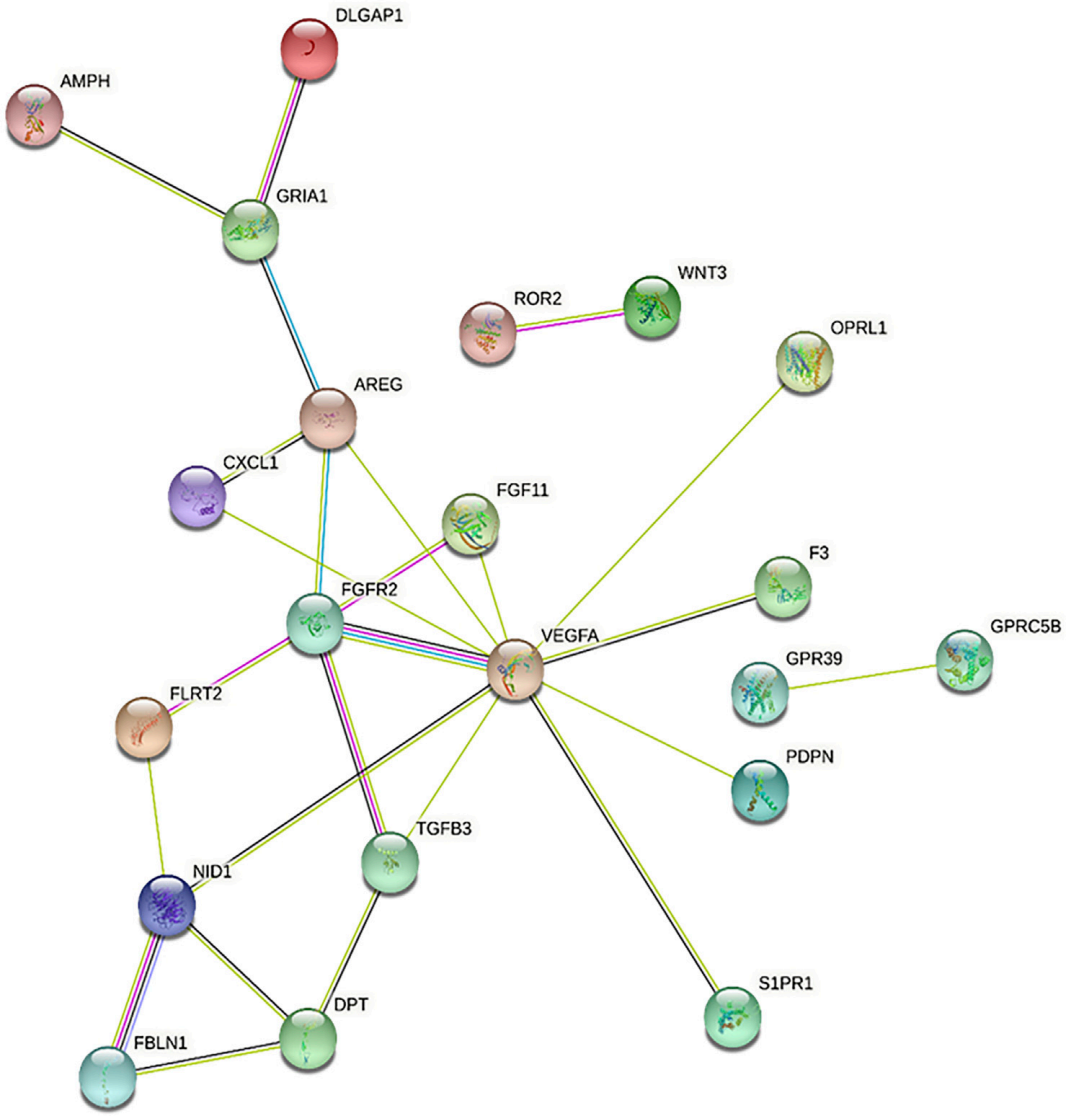
Protein-protein interaction network among genes downregulated by 1nM T3, as predicted using STRING. Genes that are central to the network, such as *VEGFA*, *FGFR2* and *TGFB3*, are associated with biological processes enriched by this treatment, as shown in Table 2.

As a second approach for identifying BPs associated with both T3 treatments, we performed PPI analysis using STRING, which is complemented by a GO term enrichment analysis based on predicted interactions (Figure 4 and Supplementary Material). In accordance with ClusterProfiler results, several of the genes downregulated by 1 nm T3 (see Table 2) were also associated with enriched terms and showed interactions with each other at the protein level (Figure 4). These genes enriched terms such as *positive regulation of Wnt signaling pathway* (GO:0030177), *wound healing* (GO:0042060) and *chemotaxis* (GO:0006935), for instance. Interestingly, also for genes upregulated by 10 nm T3, PPI analysis pointed to enriched terms related to osteoblast differentiation, namely, *negative regulation of pathway-restricted SMAD protein phosphorylation* (GO:0060394) and *regulation of BMP signaling pathway* (GO:0030510), which will be discussed below.

## Discussion

Given our current knowledge about the importance of THs for bone development and maintenance (Bochukova et al., 2012; Waung et al., 2012), several *in vitro* studies have demonstrated the effects of T3 on the expression of osteoblast markers and its modulation of bone cell metabolism (Kim and Mohan, 2013; Williams, 2013; Olímpio et al., 2019). However, the roles played by T3 in osteoblast differentiation, proliferation, development and bone formation remain unclear (Harvey et al., 2002). Considering the importance of cellular models for the study of osteoblasts, we applied an osteoinduction protocol for the differentiation of hASCs, using the cocktail previously established by our research group (Olimpio et al., 2018), and then assessed the effects of 1 and 10 nm T3 doses on the global transcriptome of osteoblast-like cells.

Our results confirm the responsiveness of hASC-derived osteoblast-like cells to T3. Several osteoblast lineages have been previously shown to respond to T3 (Klaushofer et al., 1995; Harvey et al., 2002; Waung et al., 2012; Kim and Mohan, 2013; Wojcicka et al., 2013; Olímpio et al., 2019), and the present study likewise shows that T3 at both tested doses affected genes related to bone metabolism, in BPs such as *mesenchymal cell proliferation* and *ossification*, thus confirming this human primary cell line as a suitable experimental model. Overall, it was noticeable that both T3 doses had similar effects on a subset of the DEGs, but that was not the case for the biological processes affected, which were markedly different.

The osteoblast differentiation and maintenance processes can be regulated by both mechanical and biochemical pathways (Wittkowske et al., 2016), and here we show effects of T3 on the latter. With regard to the 1 nm T3 effects, the downregulated expression of several genes related to cell differentiation and proliferation, chemotaxis, and ossification, found in this study is in agreement with the decrease of mineralized matrix formation found in our previous work (Olímpio et al., 2019). For this dose, the genes involved, summarized in Table 2, are related to osteoblast differentiation through the BMP and WNT pathways, as discussed below.

Transforming growth factor beta (TGF-β) and members of its superfamily, such as BMPs and growth/differentiation factors (GDFs), exert their effects by activating the serine/threonine kinases type I and II receptor complex, which initiates Smad-dependent or -independent intracellular signaling. Smad-dependent signaling involves the phosphorylation of R-Smads (Smads 2/3 for TGF-β and Smads 1/5/8 for BMPs/GDFs), which form complexes with Co-Smads (Smad4) that then translocate to the nucleus to activate transcription factors. Smad-independent signaling involves molecules of the mitogen-activated protein kinase (MAPK) pathways, such as extracellular signal-regulated kinases (ERKs), c-Jun N-terminal kinases (JNK), and p38. The Smad-dependent BMP pathway is known to be regulated by the inhibitory Smads (I-Smads) Smad 6 and 7, which act to suppress the pathway (Blair et al., 2002; Huang et al., 2007; Yan et al., 2009; Chen et al., 2012). Moreover, although the structure of Smad 9 (also known as Smad 8) matches that of an R-Smad, recent studies have shown that its inhibition of the BMP pathway occurs through mechanisms distinct from those of the I-Smads (Tsukamoto et al., 2015; Salazar et al., 2016).

In this context, the products of the fibroblast growth factor receptor 2 (*FGF2R*), sphingosine-1-phosphate receptor 1 (*SP1R1*), fibulin-1 (*FBLN1*), and oxidative stress responsive kinase 1 (*OSR1*) genes, which were all downregulated by 1nM T3, act synergistically on the BMP pathway to promote cell differentiation and osteoblastic function. *FGF2R* increases the expression of BMP receptor type 1B and, consequently, the effects of BMP-2, which phosphorylates the R-Smads and induces the activity of alkaline phosphatase (Singhatanadgit et al., 2006). SP1R1 enhances the BMP-2-promoted phosphorylation of ERK 1/2 and R-Smads (Sato et al., 2012). Fibulin-1, an extracellular matrix glycoprotein encoded by the *FBLN1* gene, interacts physically with BMP-2 and is necessary for the transcriptional activation of the osteogenic lineage marker Osterix and alkaline phosphatase (Cooley et al., 2014). Finally, the *OSR1* gene encodes a serine/threonine protein kinase which regulates downstream kinases and can increase the expression of BMP-4 and RUNX2 (Karvande et al., 2018).

The downregulation of vascular endothelial growth factor A (*VEGFA*) gene expression by 1 nm T3 also reinforces the conclusion that this TH dose hampers cell differentiation and proliferation, given that literature describes this gene product as being the most abundant member of the VEGF family, stimulating osteogenesis by participating in the final phases of osteoblast differentiation, in addition to acting on cell migration and proliferation (Yang et al., 2012; Hu and Olsen, 2016). It is also known that BMPs can stimulate VEGF expression in osteoblasts, promoting bone formation and angiogenesis during bone development (Deckers et al., 2002; Zhang et al., 2009).

Aside from being part of the TGF-β family and associated with the BMP pathway and bone formation, TGF-β3 has been demonstrated in a few studies to be involved in bone development as well (Chen et al., 2012; Wu et al., 2016). In this study, it was downregulated by 1 nm T3 and was associated, for instance, with the BP term *ossification*.

As mentioned above, 1 nm T3 also regulated the WNT canonical and non-canonical signaling pathways. The canonical WNT pathway depends on the activity of β-catenin as a transcription factor and plays an important role in bone metabolism. The binding of WNT proteins to their transmembrane receptor Frizzled and co-receptors lipoprotein receptor-related proteins 5 and 6 (LRP5/6) inhibits the degradation of β-catenin, which then accumulates in the cytoplasm (Baron and Rawadi, 2007) and translocates to the nucleus where it affects *RUNX2* gene transcription and promotes osteoblast differentiation and bone formation (Gaur et al., 2005; Li et al., 2005). By contrast, the non-canonical WNT pathway is independent of β-catenin and involves the activation of JNK (cellular polarity pathway) or nuclear factor of activated T-cells (NFAT) (Wnt/Ca^2+^ pathway) instead, either of which leads to the transcriptional activation of osteoblastic target genes (Enomoto et al., 2009; Gregory et al., 2010; Bretón-Romero et al., 2016).

The *WNT5A*, *WNT3*, and tyrosine-protein kinase transmembrane receptor ROR2 (*ROR2*) genes, which were downregulated by 1nM T3, play important roles in bone metabolism. WNT5A, a member of the WNT family of soluble ligands, interacts with its receptor ROR2 on the cell surface, triggering the non-canonical WNT cell polarity pathway (WNT/JNK). By contrast, WNT3 activates the canonical WNT pathway by binding to Frizzled and LRP5/6 (Gaur et al., 2005; Li et al., 2005). These genes are involved in osteoblast differentiation and proliferation, bone mineralization, and cell migration (Nishita et al., 2006; Enomoto et al., 2009; Sebastian et al., 2017), as previously shown in the pre-osteoblastic cell lines MC3T3 and SaOS-2, differentiated human mesenchymal stem cells, and mouse bone cell cultures (Liu et al., 2007a; Liu et al., 2007b).

The regulation of chemotaxis (*via FGFR2*, *S1PR1*, *OSR1*, and *VEGFA*), WNT signaling pathways (*via WNT5A*, *WNT3*, *ROR2*), and cellular responses to retinoic acid by 1 nm T3 are corroborated by the literature for *FGFR2* (Kim et al., 2007), *S1PR1* (Garnero, 2014), *VEGFA* (Yang et al., 2012; Hu and Olsen, 2016), and the WNT pathway (Gaur et al., 2005; Li et al., 2005) but not for *OSR1*. Moreover, although previous studies have demonstrated that retinoic acid is involved in osteogenic differentiation, proliferation, and mineralization and is related to the BMP and WNT pathways (Blum and Begemann, 2015; Draut et al., 2019; Roa et al., 2019), there are no published studies on its role in osteoblast migration, which could be a potential target for future study.

The *PRKCZ* gene, also downregulated by 1nM T3, encodes the atypical protein kinase C-zeta (PKCζ) from the PKC family. These proteins are described in the literature as being associated with various cell types and cellular processes, with recent studies showing their exact functions and associations with several diseases (Gopalakrishna and Jaken, 2000; Kang, 2014). However, there are as yet no studies describing the occurrence of PKCζ in osteoblasts, albeit three studies on global data have indicated its association with osteoporosis and osteosarcoma diseases (Du et al., 2014; Zhang et al., 2019; Zhou et al., 2020). Such data suggest that this molecule could be a potential biomarker for bone tissue and bone-related pathologies and is therefore worthy of further study.

In its turn, as shown by STRING Gene Ontology analysis, 10 nm T3 enriched the regulation of the BMP signaling pathway (GO: 0030510) by upregulating suppressive genes, such as *SMAD6*, *NOG*, *NEO1*, and *ENG*. This treatment enriched the *negative regulation of pathway-restricted SMAD protein phosphorylation* (GO:0060394), by increased expression of *SMAD6*, *NOG*, and *ENG*. According to the literature, Smad 6, NOG, and NEO1 inhibit the action of BMP. BMP acts by phosphorylation of the Smad proteins and is known to be regulated by the inhibitory Smads (I-Smads) Smad 6 and Smad 7, which act to suppress the pathway. NOG is an antagonist linker that binds to BMP receptors (Huang et al., 2007; Chen et al., 2012) and is a critical regulator of BMP activity during skeletogenesis and joint formation (Shi and Massagué, 2003). Neogenin 1, the protein encoded by *NEO1*, is a netrin receptor, considered to be a suppressor of BMP signaling (Abdullah et al., 2021). In addition, studies demonstrate that neogenin acts as a receptor for BMPs, and the signal transduction negatively regulates BMP-induced osteoblastic differentiation (Hagihara et al., 2011).

On the other hand, *ENG* encodes a transmembrane glycoprotein that operates as a co-receptor to the TGF-β receptor family to activate the BMP pathway by as-yet-unknown mechanisms and is involved in BMP-induced osteogenic differentiation (Ishibashi et al., 2010; Wang et al., 2014). Our results demonstrate that increased expression of the *ENG* gene enriched the negative regulation of pathway-restricted Smad protein phosphorylation. In this way, *ENG* could participate in the inhibition of an I-Smad to favor the osteogenic differentiation.

Additionaly, 10 nm T3 enhanced the expression of BMP/Smad target genes such as the *ID1* gene, which is usually upregulated following BMP-induced osteogenic stimulation and its transcription is downregulated by TGF-β (Kang et al., 2003). The role of *ID1* in osteoblast differentiation has not yet been clarified. Previous studies showed that during osteogenesis, the expression of *ID1* is initially elevated to support the proliferation of progenitor cells and then is downregulated during terminal osteoblast differentiation (Peng et al., 2004), and overexpressing *ID1* can stimulate osteoclast differentiation (Yuen et al., 2010).

Therefore, our results suggest that 10 nm T3 affects bone metabolism, by increasing the expression of genes that inhibit the BMP pathway and possibly increasing osteoclastogenesis. These results are in accordance with previous studies by our group and others, in which 10 nM T3 induced the expression of RANKL mRNA in (Miura et al., 2002; Olímpio et al., 2019) and the levels of *OPG* mRNA and protein decreased, which can favor RANKL binding to its receptor, activating osteoclastogenesis, and has a negative effect on net bone matrix formation. (Li et al., 2000; Olímpio et al., 2019).

It should be noted that other studies support a stimulating role for T3 on osteoblast differentiation and bone mineralization. Two studies (Boeloni et al., 2009; Cheng et al., 2016), found T3 to increase markers of osteoblast differentiation and ossification at doses similar to those we used. However, in the study by Boeloni and co-workers, maximum results were obtained with 10 pM T3, while most variables remained unchanged at 1 nm, which we consider to be closer to a physiological dose based on previous studies (Saraiva et al., 2008; Olímpio et al., 2019). Likewise, in two other studies (Chen et al., 2020; Yi et al., 2020), a 100 nm T3 dose was used for most experiments, which is well above our maximum 10 nm dose. Perhaps more important, these two studies were performed with cells from fetal origins, while we used mesenchymal stem cells obtained from adult donors, which could explain the different responses observed, since thyroid hormones may play different roles in early development and adulthood. Besides, all of these works used mouse or rat-derived cells, while the present study employed cells from human origin. These discrepancies underscore the need to address, in future studies, whether context-dependent shifts in osteoblast response to T3 indeed occur.

It has already been established that T3 stimulates the expression of several differentiation markers (Klaushofer et al., 1995; Varga et al., 1997; Harvey et al., 2002; Waung et al., 2012; Kim and Mohan, 2013; Wojcicka et al., 2013). The technique used in this study was not able to detect some of the genes that are recognized to be expressed in fully differentiated osteoblasts, such as *SP7* (Osterix), *TNFSF11* (RANKL), and *BSP* (bone sialoprotein). However, in a previous study (Olimpio et al., 2018), we had detected these genes by RT-qPCR, demonstrating that the osteoinduction methodology used ensures osteoblast-like differentiation. We believe that these gene transcripts have remained below the detection limit of the RNA-Seq technique. Nonetheless, to the best of our knowledge, there are no published studies on the activity of T3 in osteoblasts, making the present study an innovative and unique presentation of the biological markers affected by different doses of this TH. Considering that high doses of T3 can modify bone metabolism, causing abnormalities and culminating in pathologies *in vivo*, and given the problems encountered by patients with thyroid cancer receiving thyroid-stimulating hormone suppression therapy post thyroidectomy (Hannoush and Weiss, 2016).

Our findings on the signaling pathways potentially affected by the two doses of T3 highlight some essential points T3 in osteoblast-like cell metabolism: 1) Both doses of T3 appear to negatively influence terminal cell differentiation by inhibiting signaling pathways that are relevant to osteoblast development; 2) The effects of 10 nm T3 were likely due to BMP signaling pathway inhibition through upregulation of the expression of inhibitory genes; 3) The 1 nm T3 treatment also seems to affect the BMP signaling pathway by inhibiting the synergistic expression of genes in the pathway as well as inhibiting genes essential to the canonical and non-canonical WNT signaling pathways; 4) Both doses of T3 modulated genes related to cell migration and chemotaxis, suggesting a previously unknown role of this TH in these important biological functions; and 5) Several genes and BPs that have been scarcely studied and described in osteoblasts were revealed.

## Data Availability

The data presented in the study are deposited in GEO DataSets, accession number GSE205678.
