# Supplementary figures and images for: Effects of Triiodothyronine on Human Osteoblast-Like Cells: Novel Insights From a Global Transcriptome Analysis
Source: Front Cell Dev Biol. 2022 Jun 17;10:886136. doi: 10.3389/fcell.2022.886136 (PMC9248766; doi:10.3389/fcell.2022.886136)

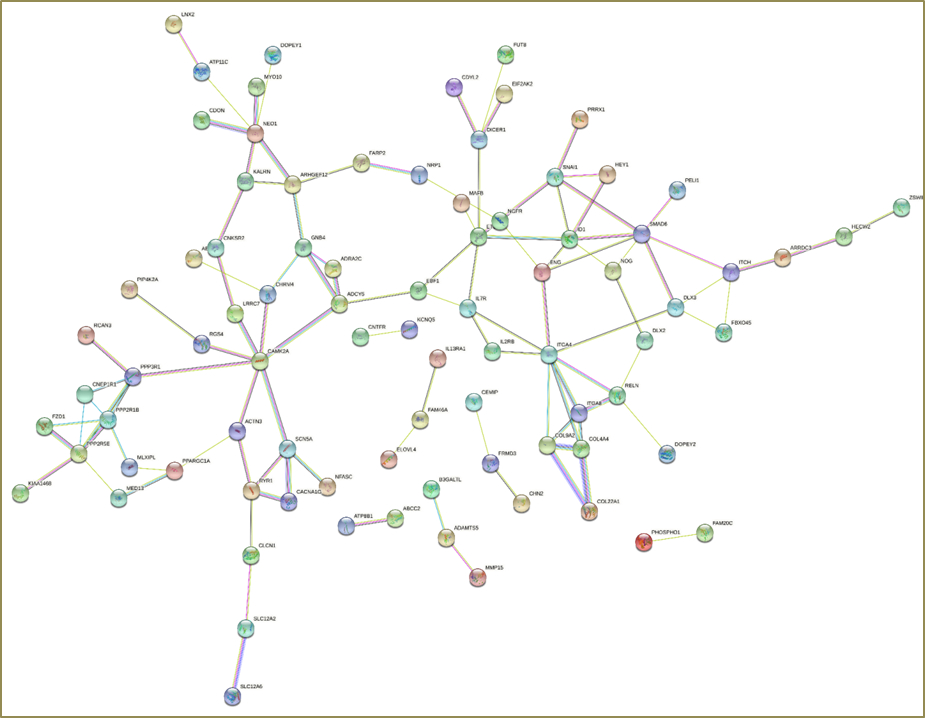

Supplement: Supplementary file 1 [file Image3.JPEG]

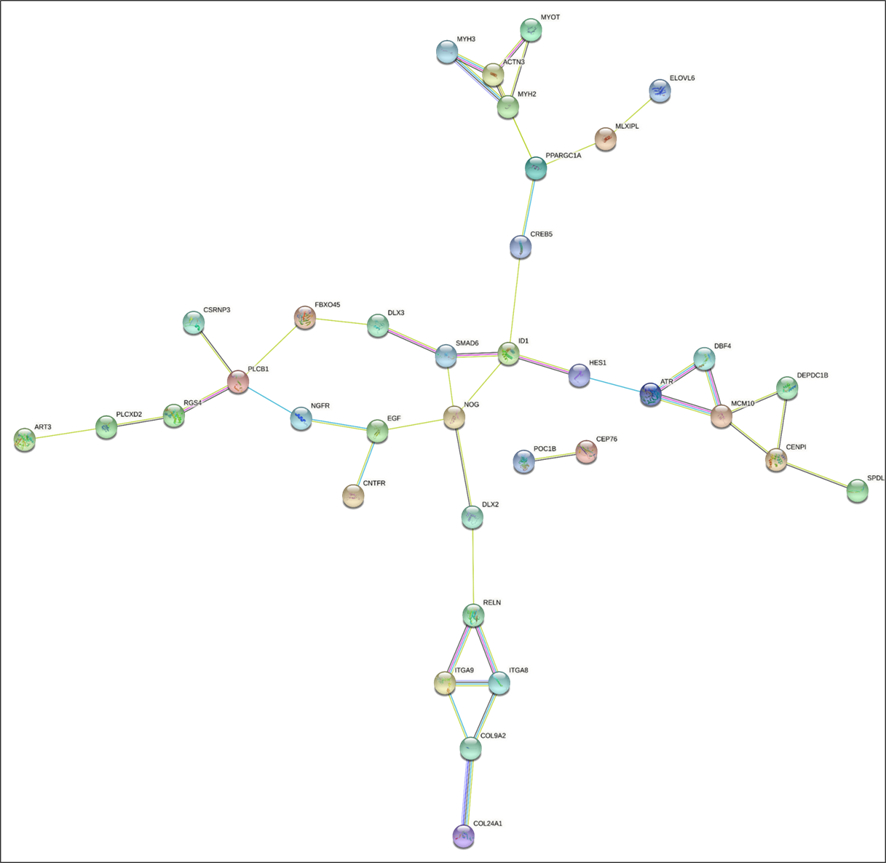

Supplement: Supplementary file 5 [file Image1.JPEG]

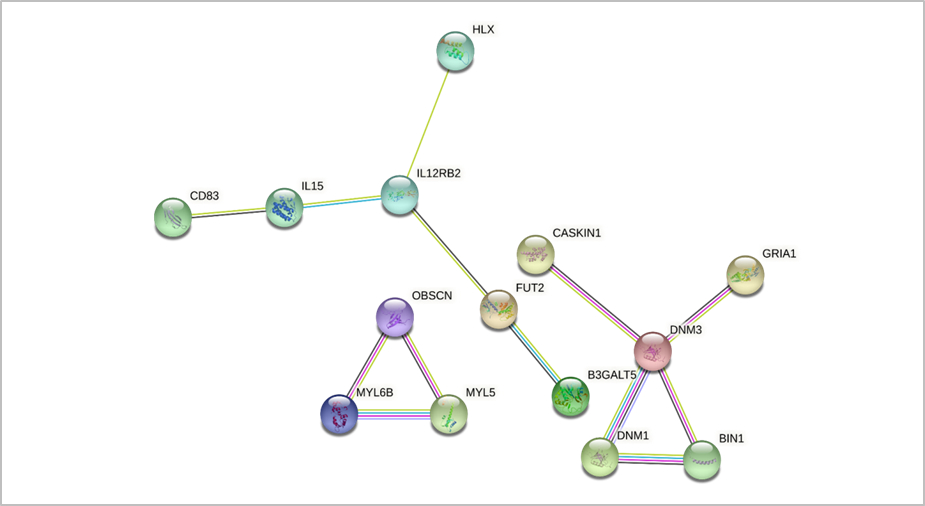

Supplement: Supplementary file 6 [file Image2.JPEG]
